# Supplementary material for: Dimer‐specific immunoprecipitation of active caspase‐2 identifies TRAF proteins as novel activators
Source: EMBO J. 2018 Jun 6;37(14):e97072. doi: 10.15252/embj.201797072 (PMC6043850; doi:10.15252/embj.201797072)
Supplement: Supplementary file 1 — Expanded View Figures PDF [file EMBJ-37-e97072-s001.pdf]

Expanded View Figures

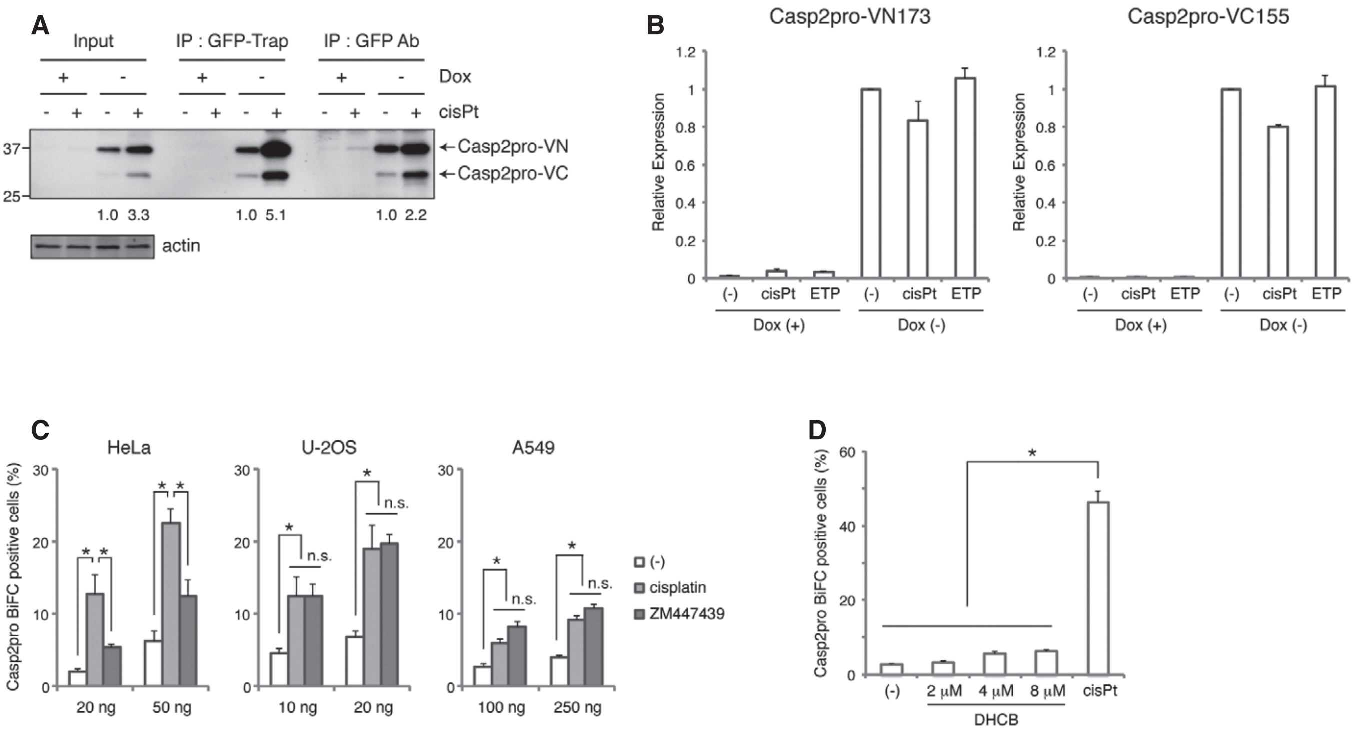

Figure EV1. GFP-Trap precipitates BiFC dimers more efficiently and with less background compared to conventional immunoprecipitation, and cisplatin is a direct inducer of caspase-2 dimerization.

A Comparison of Casp2pro BiFC dimer immunoprecipitation with GFP-Trap and conventional immunoprecipitation with anti-GFP antibody. Casp2pro BiFC cells were treated with 20 μM cisplatin in the presence of 10 μM Q-VD(OMe)-OPh for 24 h. Relative band intensities of Casp2pro-VN were quantified, and ratio was indicated (values were normalized to the relevant mock treatment). Molecular weight markers are indicated in kDa.

B mRNA expression of Casp2pro-VN173 and Casp2pro-VC155 was analyzed by qPCR after treatment of Casp2pro BiFC cells with mock, 20 μM cisplatin, or 50 μM etoposide in the presence of 10 μM Q-VD(OMe)-OPh for 24 h. $n = 3$ independent experiments (means + s.e.m.).

C Indicated amount of Casp2pro BiFC constructs was transfected into HeLa, U-2OS, or A549 cells and allowed to express for 24 h. Cells were then treated with 20 μM cisplatin or 2 μM ZM447439 in the presence of 10 μM Q-VD(OMe)-OPh for 24 h, and BiFC was detected by flow cytometry. $n = 5$ (HeLa), $n = 3$ (U-2OS), or $n = 4$ (A549) independent experiments (means + s.e.m.). *$P < 0.05$ (HeLa, U-2OS), $P < 0.01$ (A549) by unpaired two-tailed t-test. n.s.; not significant.

D Casp2pro BiFC cells were treated with the indicated concentrations of dihydrocytochalasin B (DHCB) or 20 μM cisplatin in the presence of 10 μM Q-VD(OMe)-OPh for 24 h. BiFC was detected by flow cytometry. $n = 4$ independent experiments (means + s.e.m.). $P < 0.005$ by unpaired two-tailed t-test.

Source data are available online for this figure.

© 2018 The Authors

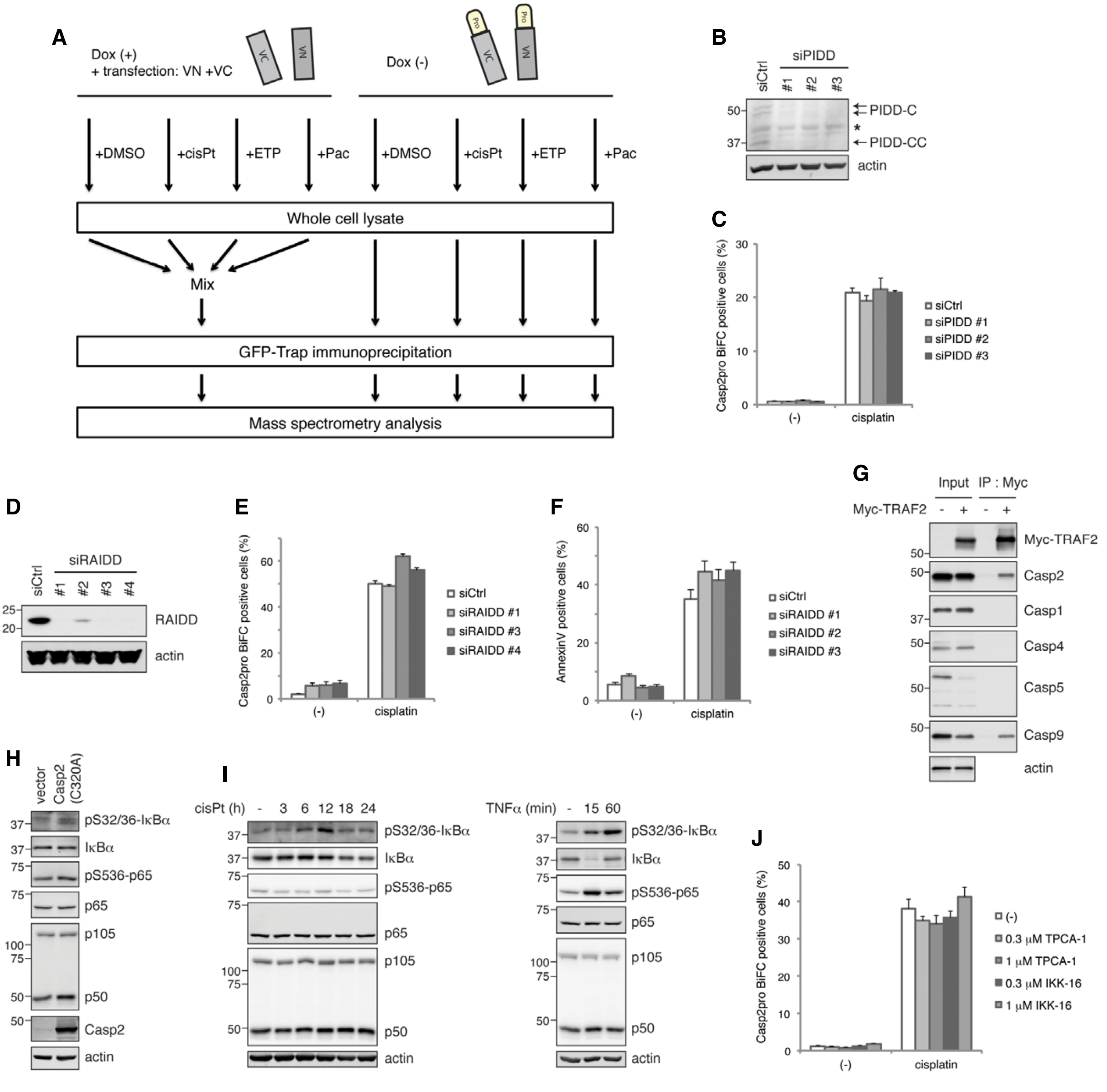

Figure EV2.

 © 2018 The Authors

Figure EV2. Workflow of mass spectrometry analysis. Cisplatin induces caspase-2 dimerization independently of either the PIDDosome or the NF-κB pathway.

A Workflow for mass spectrometry analysis. Casp2pro BiFC cells were treated with mock (DMSO), 20 μM cisplatin, 50 μM etoposide, or 100 nM paclitaxel in the presence of 10 μM Q-VD(OMe)-OPh for 24 h followed by GFP-Trap immunoprecipitation. Note the control: Casp2pro BiFC expression was prevented with 1 μg/ml doxycycline, and unconjugated BiFC constructs (VN and VC) were co-transfected into cells before treatment, similar to experimental samples. Control lysates were then pooled before GFP-Trap IP. GFP-Trap beads were subjected to on-bead protein digestion and analyzed by mass spectrometry.

B, C Casp2pro BiFC cells were transfected with siRNA targeting PIDD for 48 h, and knockdown efficiency was confirmed by IB (B). PIDD siRNA transfected cells were treated with 20 μM cisplatin in the presence of 10 μM Q-VD(OMe)-OPh for 24 h. Caspase-2 BiFC was assessed by flow cytometry. n = 3 independent experiments (means + s.e.m.) (C).

D, E Casp2pro BiFC cells were transfected with siRNA targeting RAIDD for 48 h, and knockdown efficiency was confirmed by IB (D). RAIDD siRNA transfected cells were treated with 20 μM cisplatin in the presence of 10 μM Q-VD(OMe)-OPh for 24 h. Caspase-2 BiFC was assessed by flow cytometry. n = 3 independent experiments (means + s.e.m.) (E).

F HeLa cells were transfected with RAIDD siRNA for 48 h and then treated with 20 μM cisplatin for 24 h. Apoptosis was assessed by annexin V staining and flow cytometry. n = 3 independent experiments (means + s.e.m.).

G HeLa cells were transfected with Myc-TRAF2 construct and cultured 48 h for expression. Cell lysates were prepared, followed by Myc-Trap immunoprecipitation and IB for indicated caspases.

H HeLa cells were transfected with caspase-2(C320A) construct and cultured 48 h for expression. Cell lysates were prepared, followed by IB.

I HeLa cells were treated with 20 μM cisplatin in the presence of 10 μM Q-VD(OMe)-OPh (left) or treated with 20 ng/ml TNFα (right) for indicated periods. Cell lysates were prepared and analyzed by IB.

J Casp2pro BiFC cells were treated with 20 μM cisplatin in the presence of 10 μM Q-VD(OMe)-OPh with or without indicated NF-κB pathway inhibitors, TPCA-1 or IKK-16, at indicated concentrations for 24 h. Caspase-2 BiFC was assessed by flow cytometry. n = 3 independent experiments (means + s.e.m.).

Source data are available online for this figure.

© 2018 The Authors

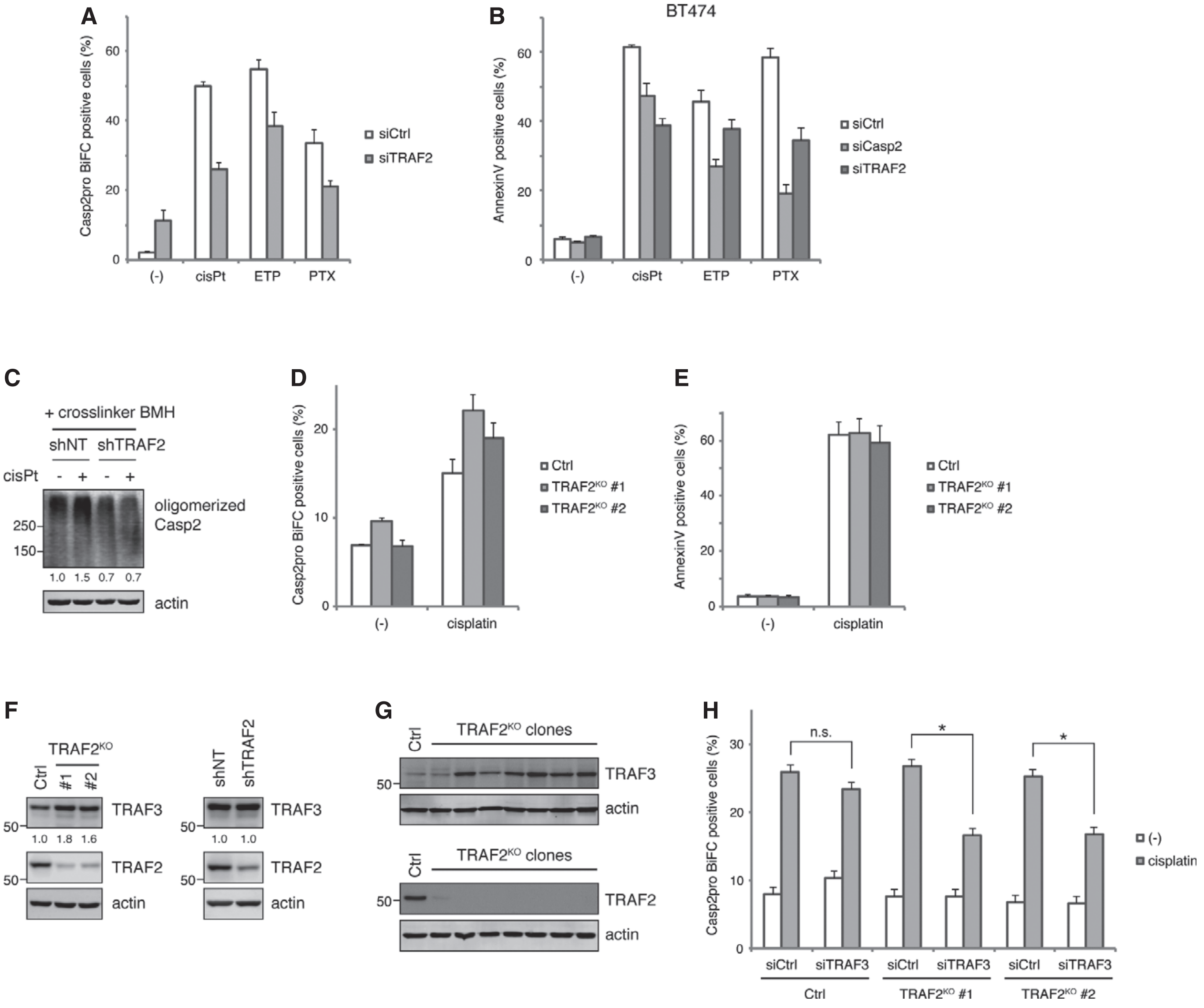

Figure EV3. TRAF2 acts as a significant regulator of caspase-2 activation across multiple scenarios, while its genetic ablation leads to compensation from TRAF3.

A Casp2pro BiFC cells were transfected with TRAF2 siRNA for 48 h and then treated with 20 μM cisplatin, 50 μM etoposide, or 100 nM paclitaxel in the presence of 10 μM Q-VD(OMe)-OPh for 24 h. Caspase-2 dimerization was assessed by flow cytometry. $n = 3$ independent experiments (means + s.e.m.).

B Caspase-2 or TRAF2 was knocked down by siRNA in BT474 breast cancer cells for 48 h, followed by treatment with 40 μM cisplatin, 100 μM etoposide, or 100 nM paclitaxel for 72 h. Apoptosis was assessed by annexin V staining and flow cytometry. $n = 4$ independent experiments (means + s.e.m.).

C shNT or shTRAF2 #1 HeLa cells were treated with 20 μM cisplatin in the presence of 10 μM Q-VD(OMe)-OPh for 24 h. Cells were collected and incubated with BMH for protein crosslinking, and then, lysates were prepared and oligomerized caspase-2 was detected by IB. Relative signal intensity of oligomerized caspase-2 was quantified and indicated below each lane (signal was normalized to mock treatment of shNT).

D TRAF2 CRISPR-KO HeLa cells were transfected with 50 ng pair of Casp2pro BiFC constructs and cultured for 24 h. Cells were treated with 20 μM cisplatin in the presence of 10 μM Q-VD(OMe)-OPh for 24 h, and caspase-2 BiFC was assessed by flow cytometry. $n = 4$ independent experiments (means + s.e.m.).

E TRAF2 CRISPR-KO HeLa cells were treated with 20 μM cisplatin for 24 h. Apoptosis was assessed by annexin V staining and flow cytometry. $n = 3$ independent experiments (means + s.e.m.).

F Lysates of TRAF2 CRISPR-KO HeLa (left) or shTRAF2 #1 HeLa cells (right) were prepared, followed by IB. Relative band intensity of TRAF3 was quantified and indicated below each lane [signal was normalized to control (left) or shNT (right)].

G Lysates of several clones of TRAF2 CRISPR-KO HeLa cells were prepared, followed by IB.

H Control or TRAF2 CRISPR-KO HeLa cells were transfected with TRAF3 siRNA, followed by Casp2pro BiFC construct transfection. Cells were treated with 20 μM cisplatin in the presence of 10 μM Q-VD(OMe)-OPh for 24 h. Caspase-2 BiFC was assessed by flow cytometry. $n = 4$ independent experiments (means + s.e.m.). *$P < 0.01$ (TRAF2^{KO} #1), $P < 0.05$ (TRAF2^{KO} #2) by unpaired two-tailed t-test. n.s.; not significant.

Source data are available online for this figure.

 © 2018 The Authors

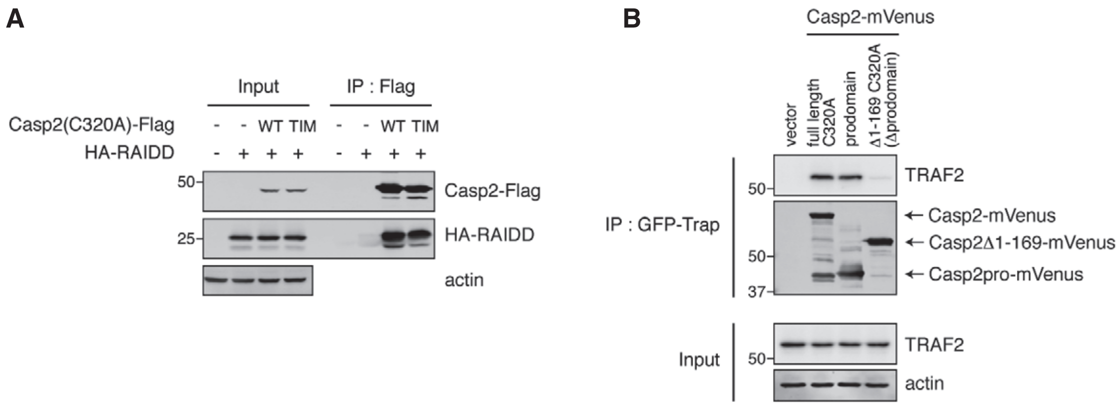

Figure EV4. Mutation of the TRAF-interacting motif of caspase-2 does not disrupt RAIDD binding, while the caspase-2 prodomain is also important for binding TRAF2.

A HEK293T cells were co-transfected with indicated caspase-2(C320A)-Flag (wild type or TIM mutant) and HA-RAIDD constructs. After 48-h culture for expression, lysates were prepared, followed by anti-Flag IP and IB.

B HEK293T cells were transfected with siRNA targeting caspase-2 3′-UTR to deplete endogenous caspase-2. 24 h post-siRNA transfection, cells were then transfected with indicated Casp2-mVenus constructs (full-length C320A, prodomain, or Δ1–169 C320A (Δprodomain)). Casp2-mVenus variants were immunoprecipitated by GFP-Trap, followed by IB for co-immunoprecipitated TRAF2.

Source data are available online for this figure.

© 2018 The Authors

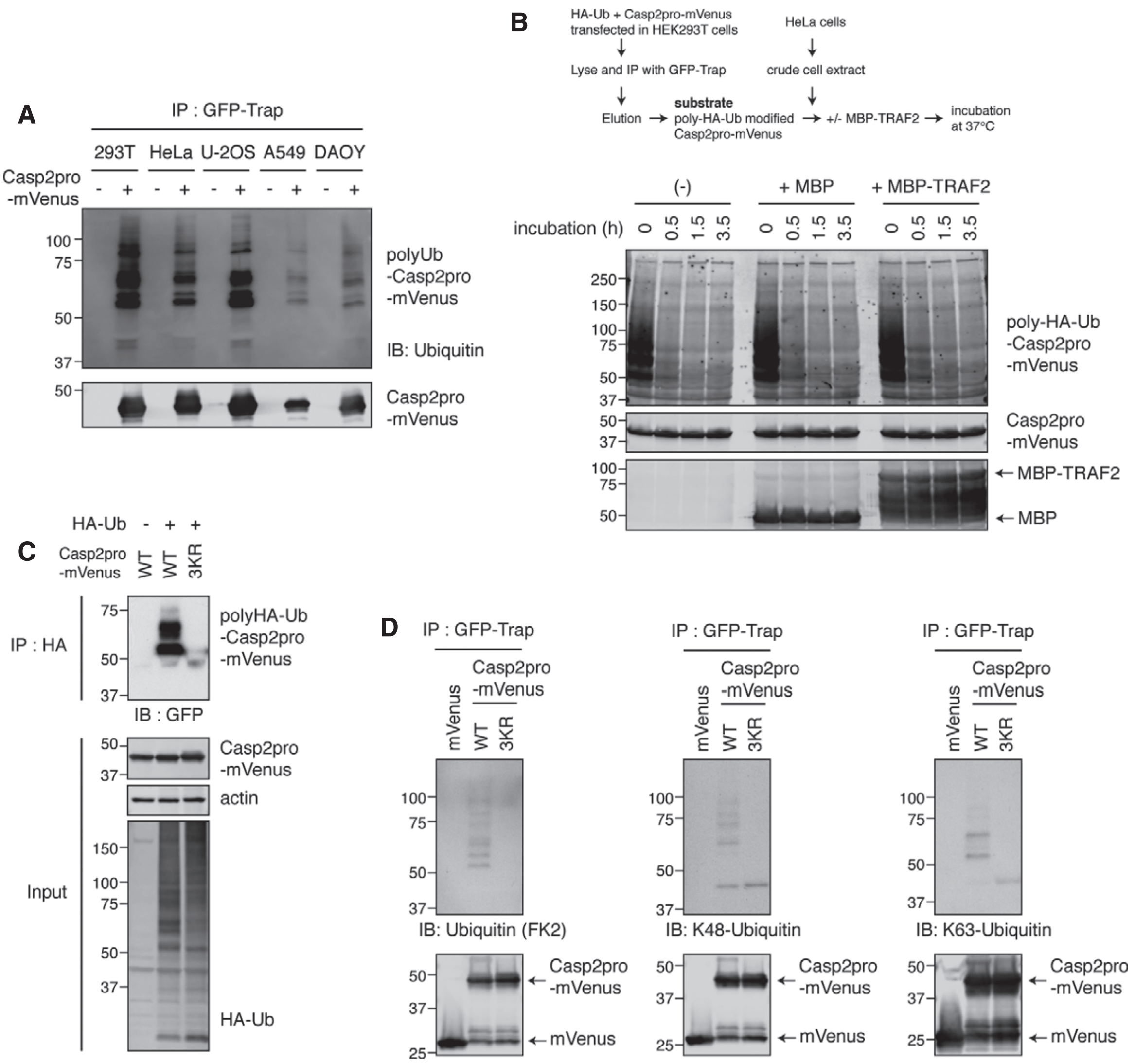

Figure EV5. TRAF2 does not regulate removal of caspase-2 ubiquitin modifications, which consist of both K48- and K63-linked chains.

A HEK293T, HeLa, U-2OS, A549, or DAOY cells were transfected with Casp2pro-mVenus construct and cultured 48 h for expression. Casp2pro-mVenus was immunoprecipitated by GFP-Trap and blotted with anti-ubiquitin antibody to detect ubiquitylated Casp2pro-mVenus.

B *In vitro* deubiquitylation assay of caspase-2. Casp2pro-mVenus was ubiquitylated with HA-ubiquitin in HEK293T cells and purified by GFP-Trap IP and elution. Then, poly-HA-ubiquitin-modified Casp2pro-mVenus was added to HeLa cell lysate with or without recombinant MBP-TRAF2 or MBP control protein. The mixture was incubated at 37°C for indicated periods and analyzed by immunoblot to assess whether TRAF2 could oppose caspase-2 deubiquitylation.

C HA-ubiquitin and Casp2pro-mVenus (wild type or 3KR mutant) were co-transfected into HEK293T cells, and lysates were immunoprecipitated by anti-HA affinity beads and analyzed by IB.

D HEK293T cells were transfected with Casp2pro-mVenus, wild type or 3KR mutant, followed by ubiquitylated Casp2pro-mVenus purification as in (A). IB was carried out with anti-ubiquitylated protein antibody (FK2), K48-linkage-specific, or K63-linkage-specific anti-ubiquitin antibody.

Source data are available online for this figure.

 © 2018 The Authors

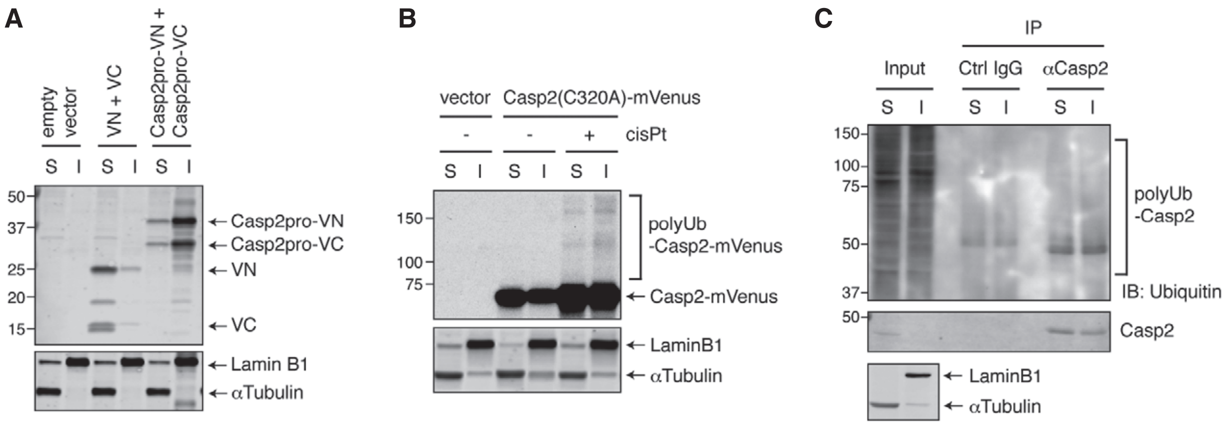

Figure EV6. Cisplatin promotes accumulation of ubiquitylated caspase-2 in the detergent-insoluble fraction.

A Caspase-2 shifts BiFC dimer localization to an insoluble fraction. Pairs of unconjugated BiFC fragments or Casp2pro BiFC fragments were overexpressed in HEK293T cells for 24 h to induce dimerization, after which cells were biochemically fractionated (S: soluble, I: insoluble fraction) and assessed by IB.

B HeLa cells were transfected with Casp2(C320A)-mVenus construct and allowed to express for 24 h and then treated with or without 20 μM cisplatin in the presence of 10 μM Q-VD(OMe)-OPh for 24 h. Cells were biochemically fractionated and assessed by IB.

C HeLa cells were treated with 20 μM cisplatin in the presence of 10 μM Q-VD(OMe)-OPh for 24 h and then biochemically fractionated. Lysates were immunoprecipitated in denaturing/renaturing conditions with anti-caspase-2 antibody or control IgG, followed by IB.

Source data are available online for this figure.

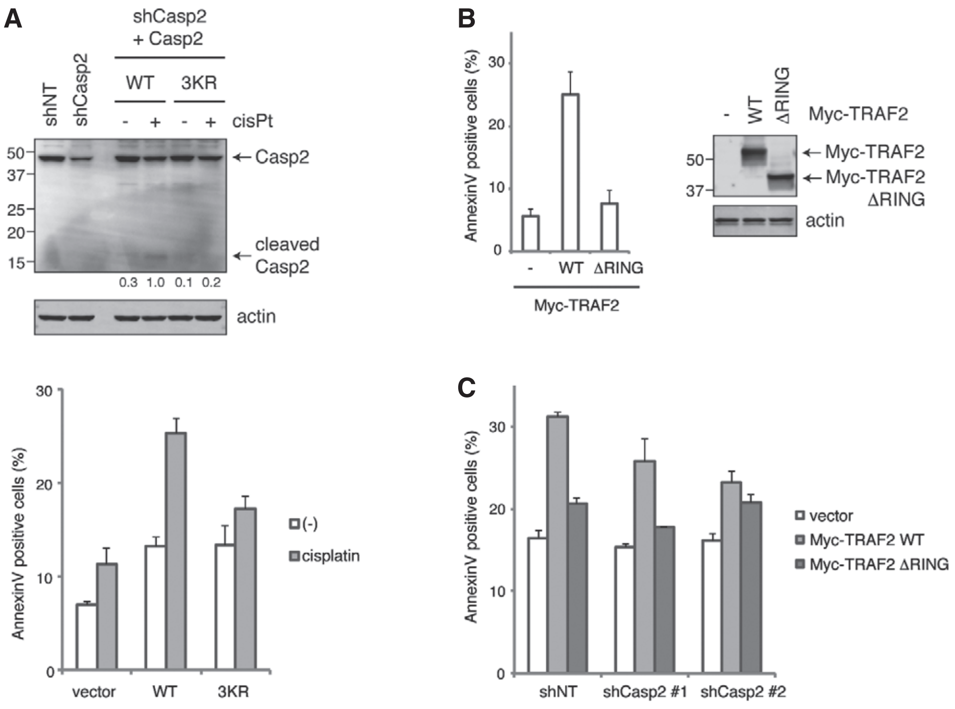

Figure EV7. TRAF2 overexpression-induced cell death requires both the RING domain and caspase-2.

A shCasp2 #1 cells were transfected with indicated caspase-2 construct (wild type or 3KR mutant) containing shCasp2-resistant sequence. Cells were treated with 10 μM cisplatin for 24 h, followed by IB. Relative band intensity of cleaved caspase-2 was quantified and indicated below each lane (signal was normalized to cisplatin treatment of WT) (top). Transfected cells were treated with 5 μM cisplatin for 24 h, and then analyzed by annexin V staining and flow cytometry. *n* = 4 independent experiments (means + s.e.m.) (bottom).

B HeLa cells were transfected with indicated Myc-TRAF2 construct (wild type or RING domain mutant) for 48 h. Apoptosis was analyzed by annexin V staining and flow cytometry. *n* = 3 independent experiments (means + s.e.m.) (left). Expression of Myc-TRAF2 was confirmed by IB (right).

C HeLa shNT, shCasp2 #1, or shCasp2 #2 cells were transfected as in (B), and apoptosis was analyzed by annexin V staining and flow cytometry. *n* = 3 independent experiments (means + s.e.m.).

Source data are available online for this figure.

 © 2018 The Authors
