# Supplementary figures and images for: Dimer‐specific immunoprecipitation of active caspase‐2 identifies TRAF proteins as novel activators
Source: EMBO J. 2018 Jun 6;37(14):e97072. doi: 10.15252/embj.201797072 (PMC6043850; doi:10.15252/embj.201797072)

Figure EV1

A

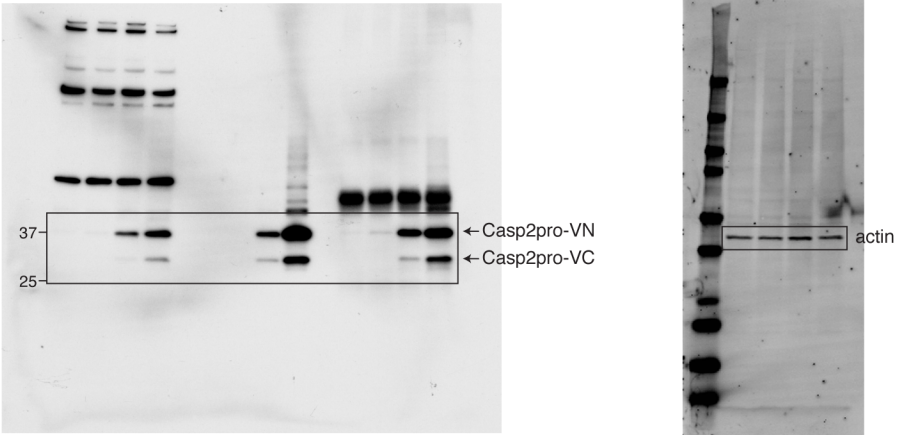

Supplement: Supplementary file 2 — Source Data for Expanded View [file EMBJ-37-e97072-s009.zip › Expanded_Figure_source_files/Figure_EV1_source_file.pdf]

Figure EV2

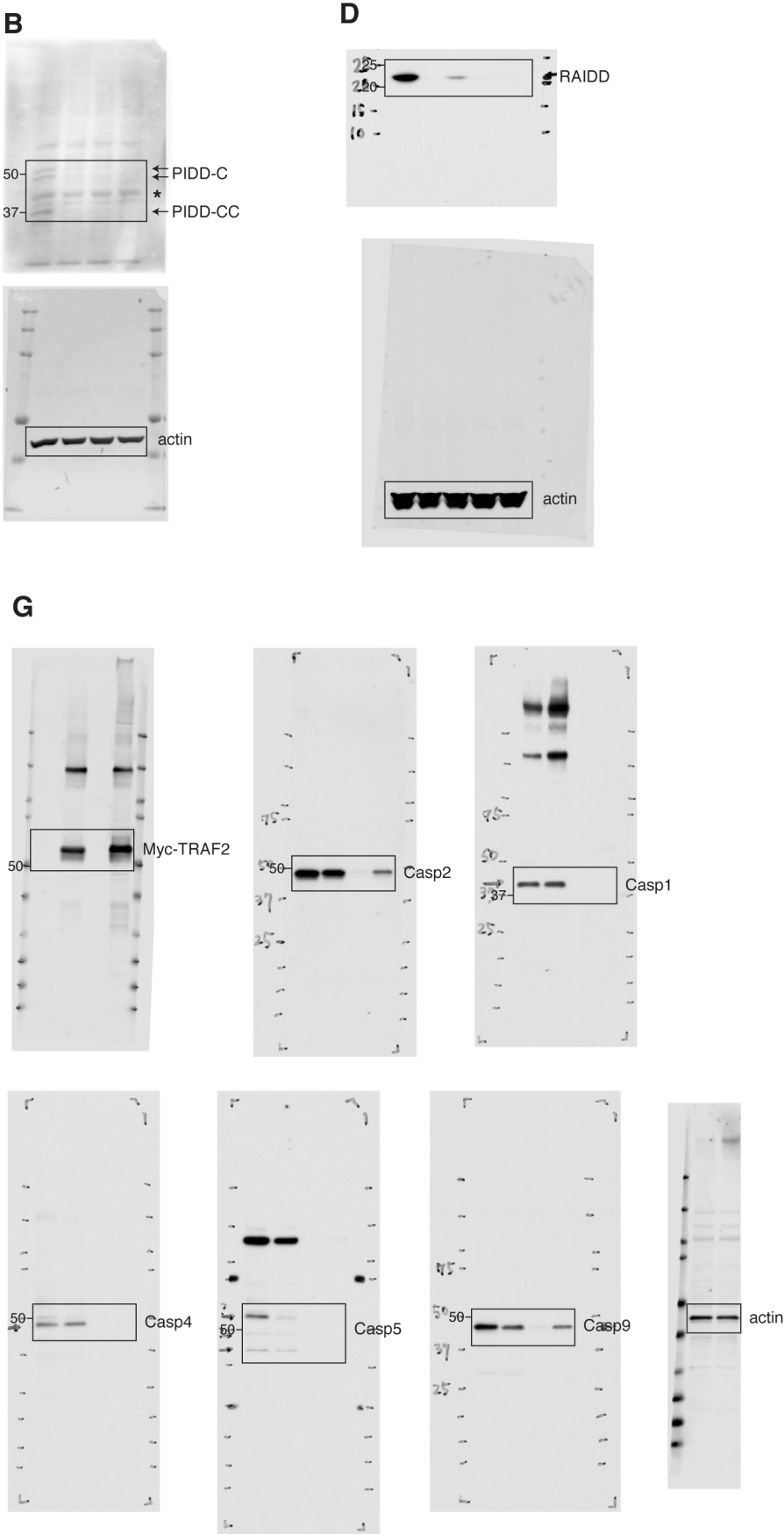

Figure EV2

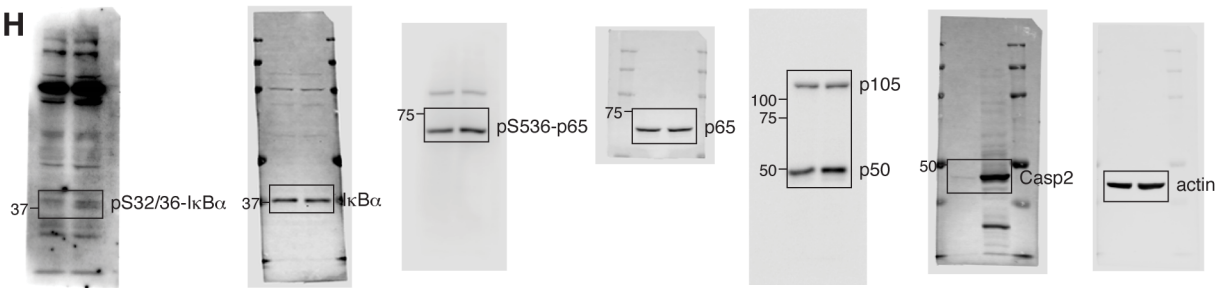

**I (left)**

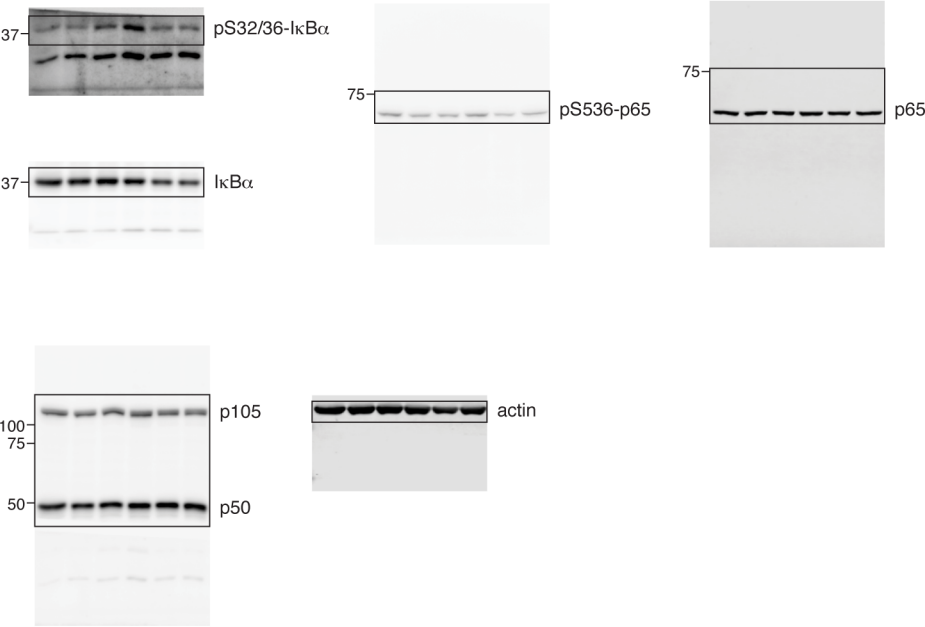

**I (right)**

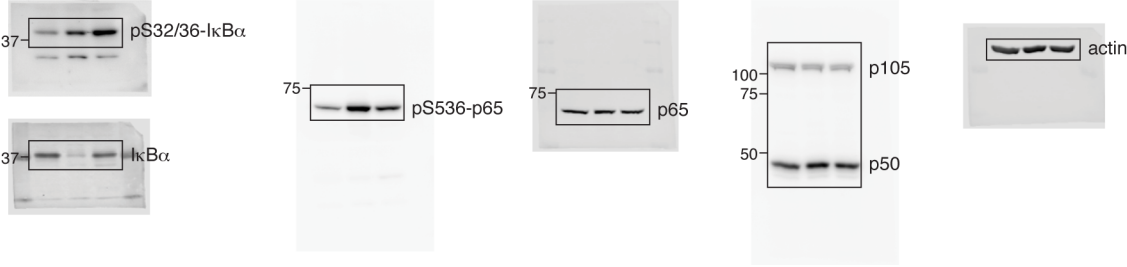

Supplement: Supplementary file 2 — Source Data for Expanded View [file EMBJ-37-e97072-s009.zip › Expanded_Figure_source_files/Figure_EV2_source_file.pdf]

Figure EV3

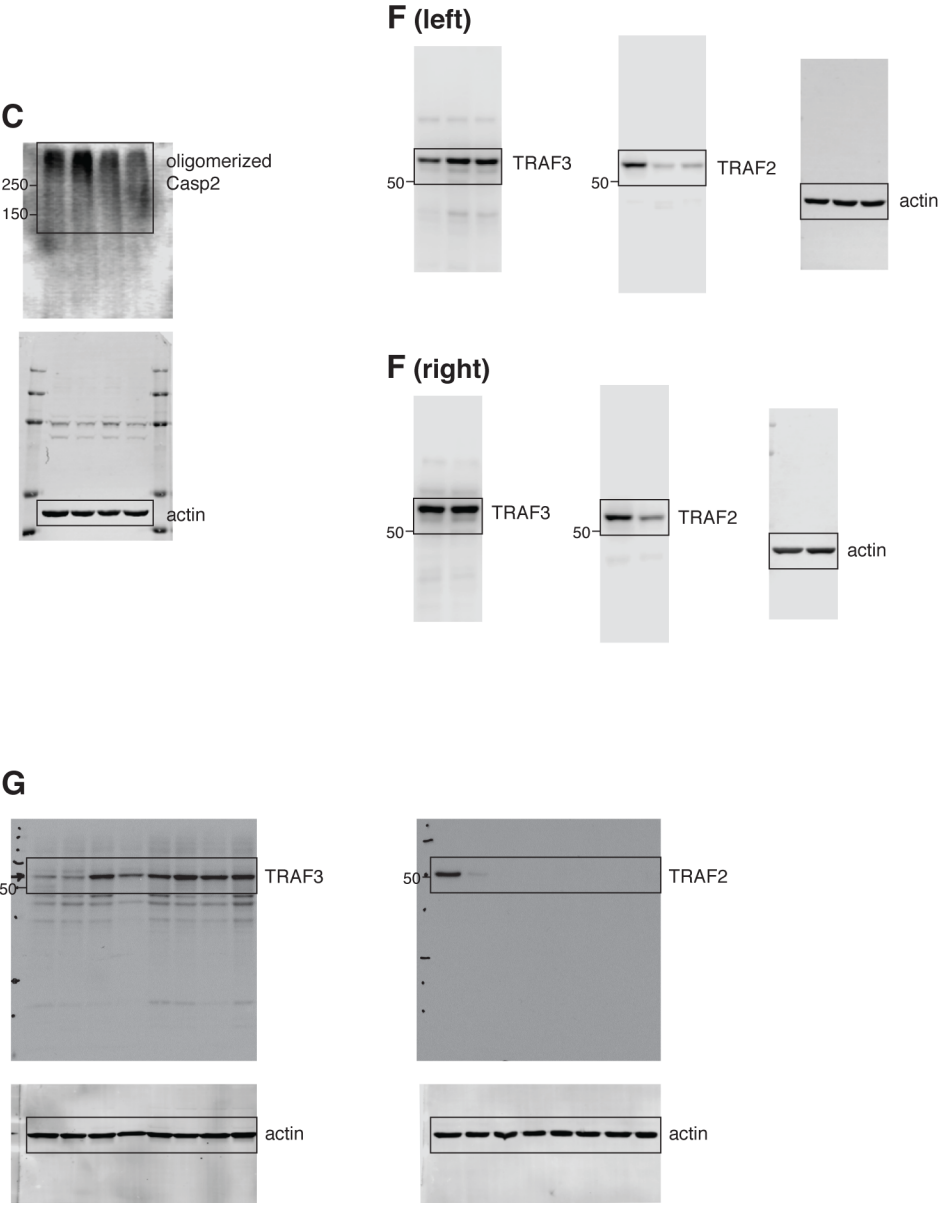

Supplement: Supplementary file 2 — Source Data for Expanded View [file EMBJ-37-e97072-s009.zip › Expanded_Figure_source_files/Figure_EV3_source_file.pdf]

Figure EV4

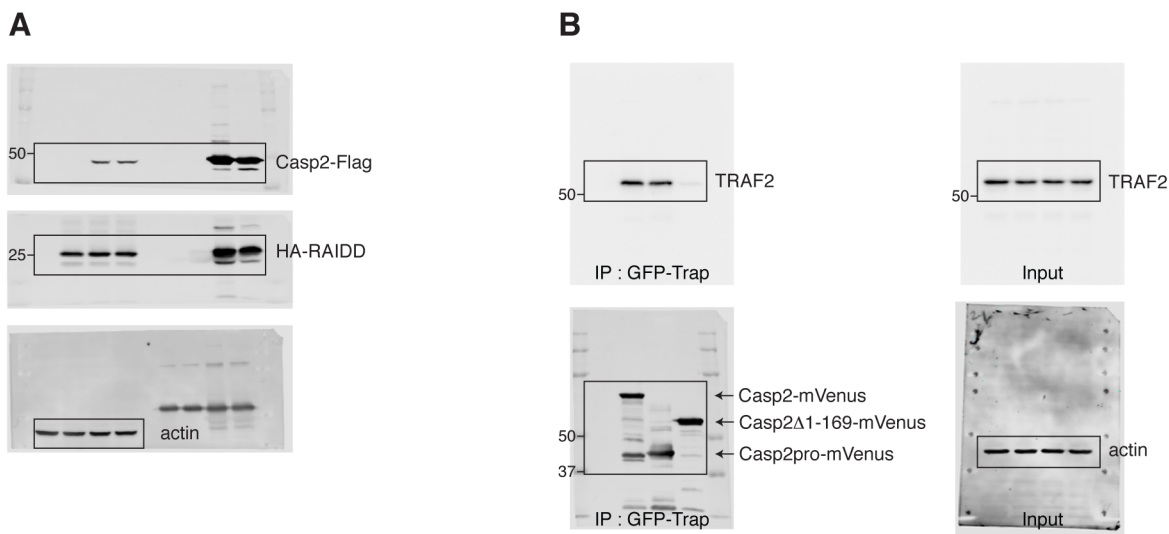

Supplement: Supplementary file 2 — Source Data for Expanded View [file EMBJ-37-e97072-s009.zip › Expanded_Figure_source_files/Figure_EV4_source_file.pdf]

Figure EV5

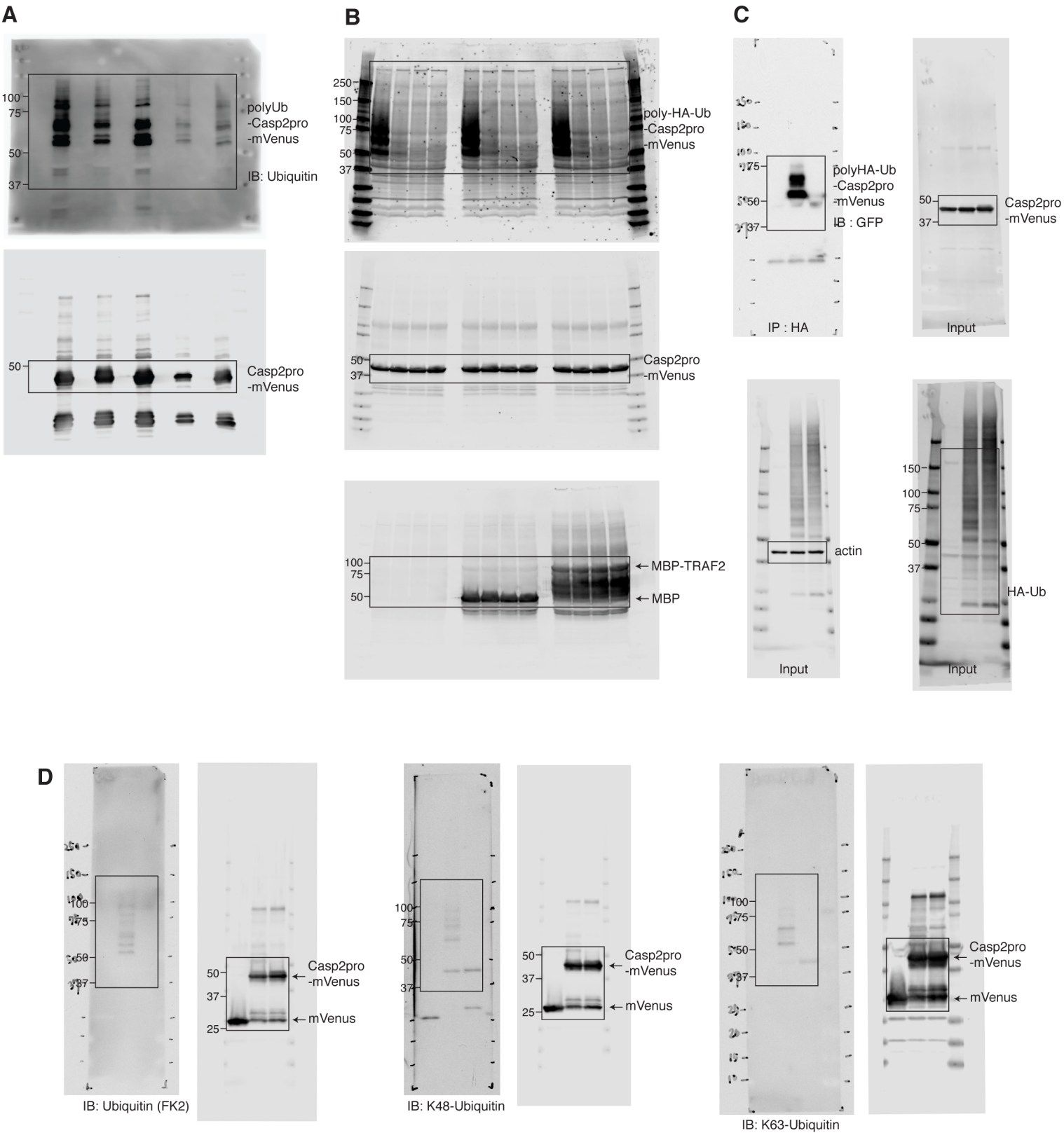

Supplement: Supplementary file 2 — Source Data for Expanded View [file EMBJ-37-e97072-s009.zip › Expanded_Figure_source_files/Figure_EV5_source_file.pdf]

Figure EV6

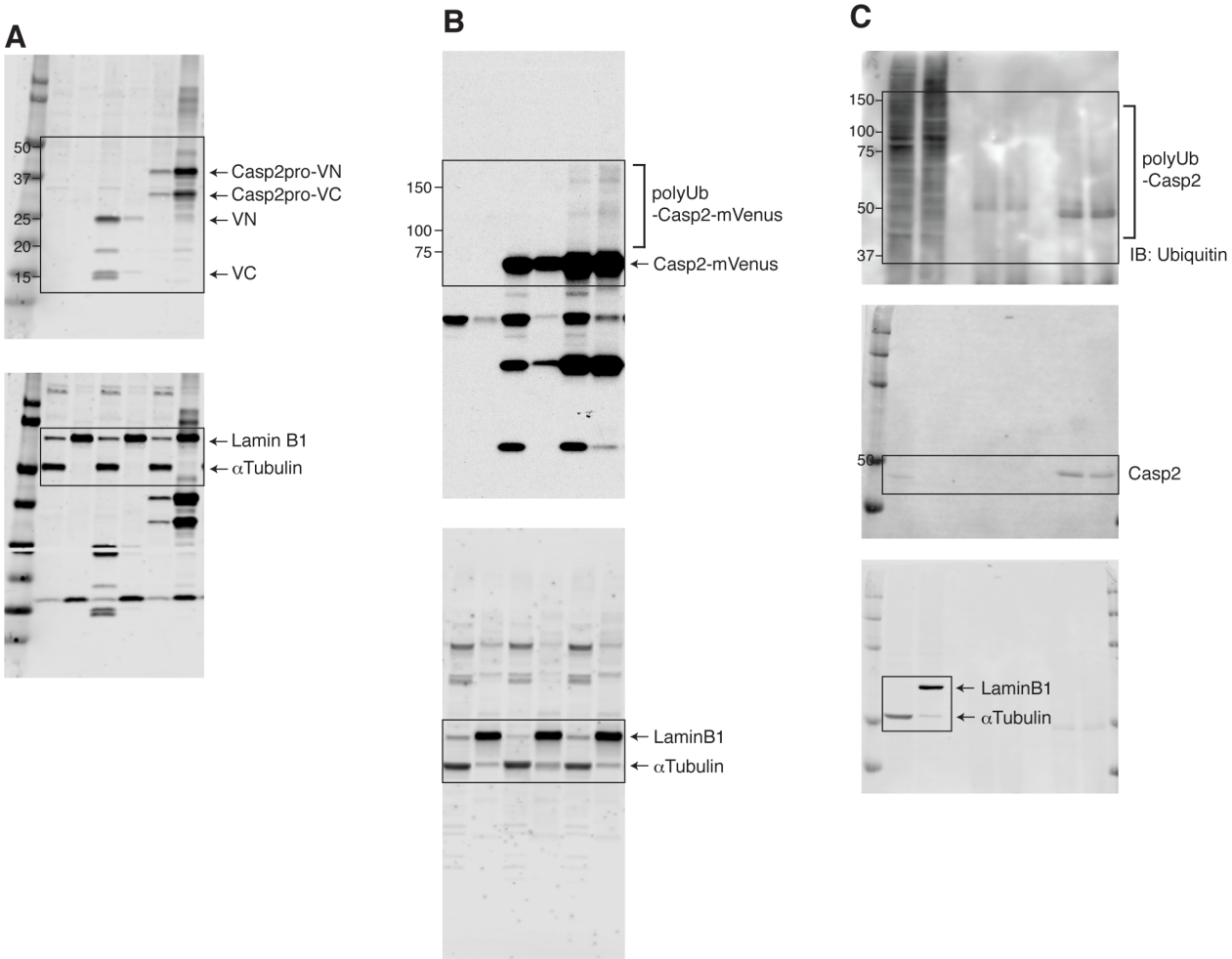

Supplement: Supplementary file 2 — Source Data for Expanded View [file EMBJ-37-e97072-s009.zip › Expanded_Figure_source_files/Figure_EV6_source_file.pdf]

Figure EV7

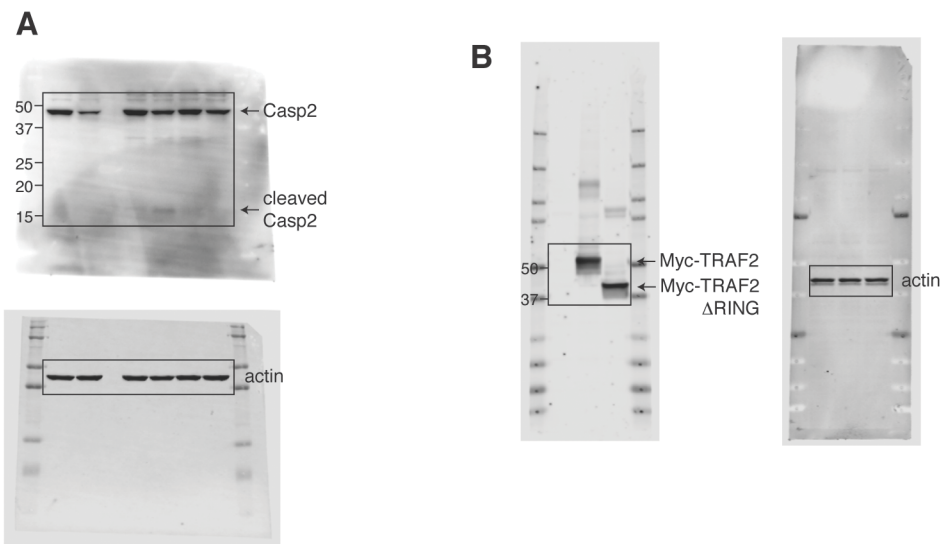

Supplement: Supplementary file 2 — Source Data for Expanded View [file EMBJ-37-e97072-s009.zip › Expanded_Figure_source_files/Figure_EV7_source_file.pdf]

Figure 1

B

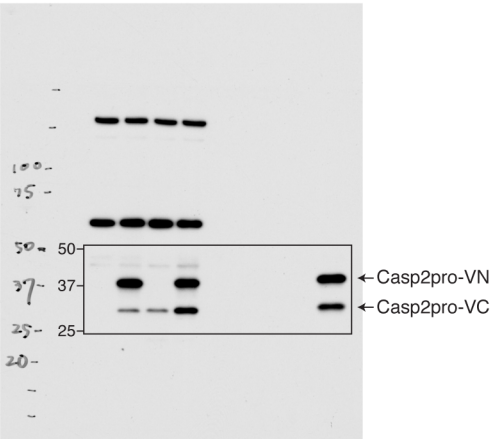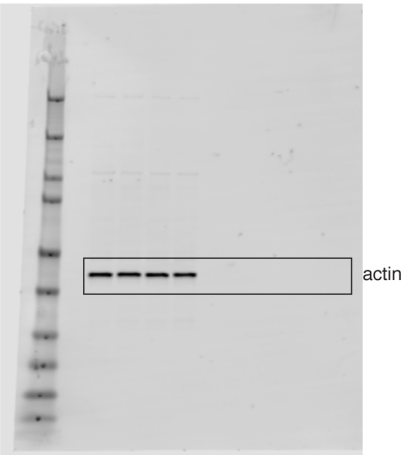

E

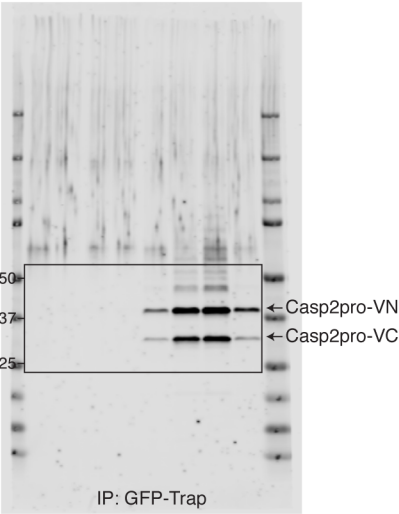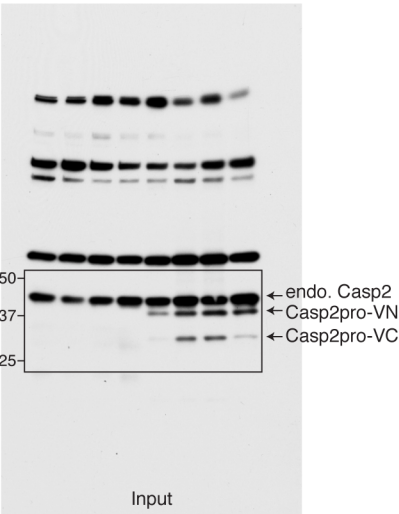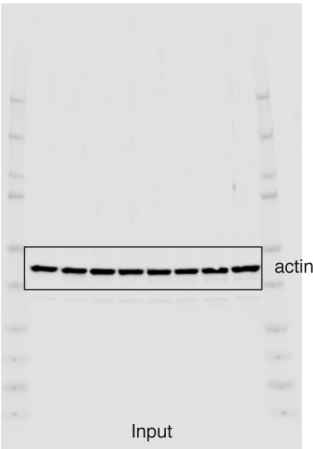

Supplement: Supplementary file 4 — Source Data for Figure 1 [file EMBJ-37-e97072-s002.pdf]

Figure 2

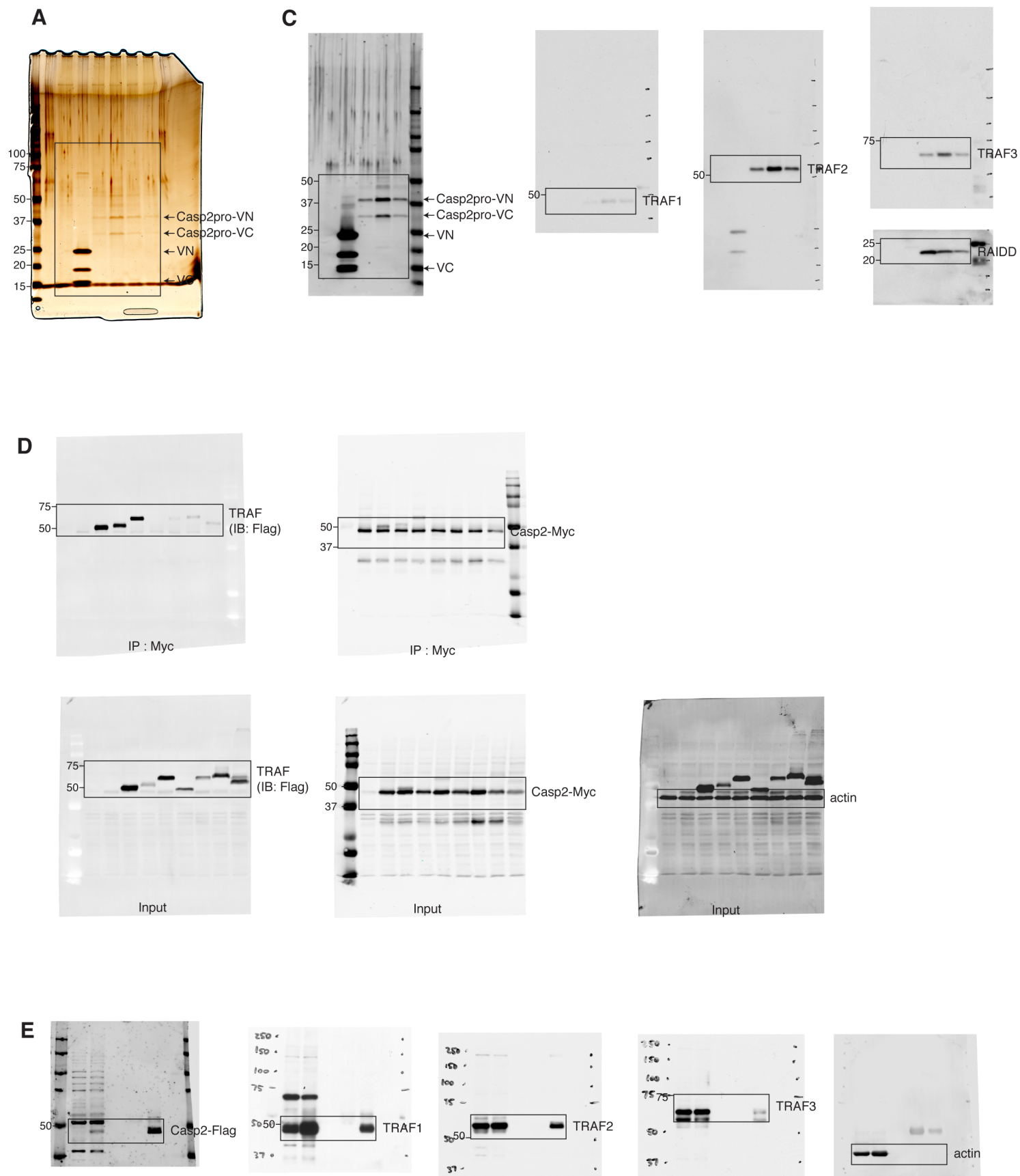

Figure 2

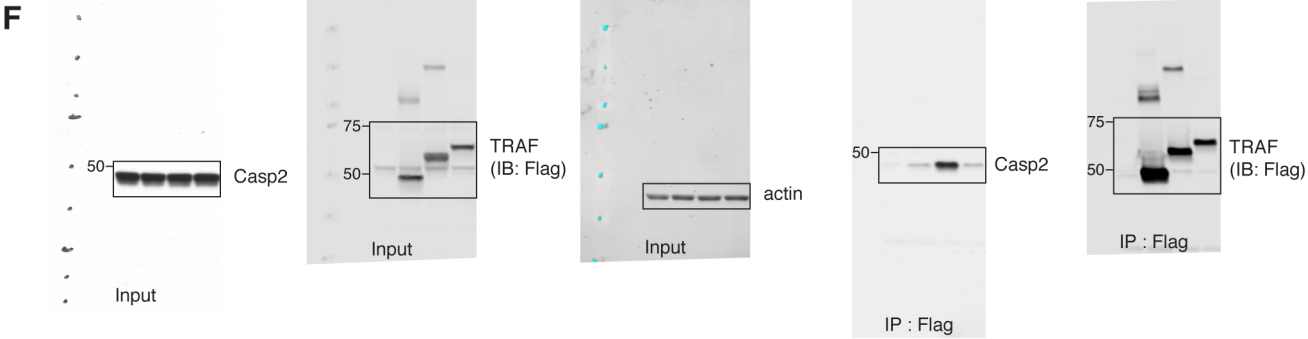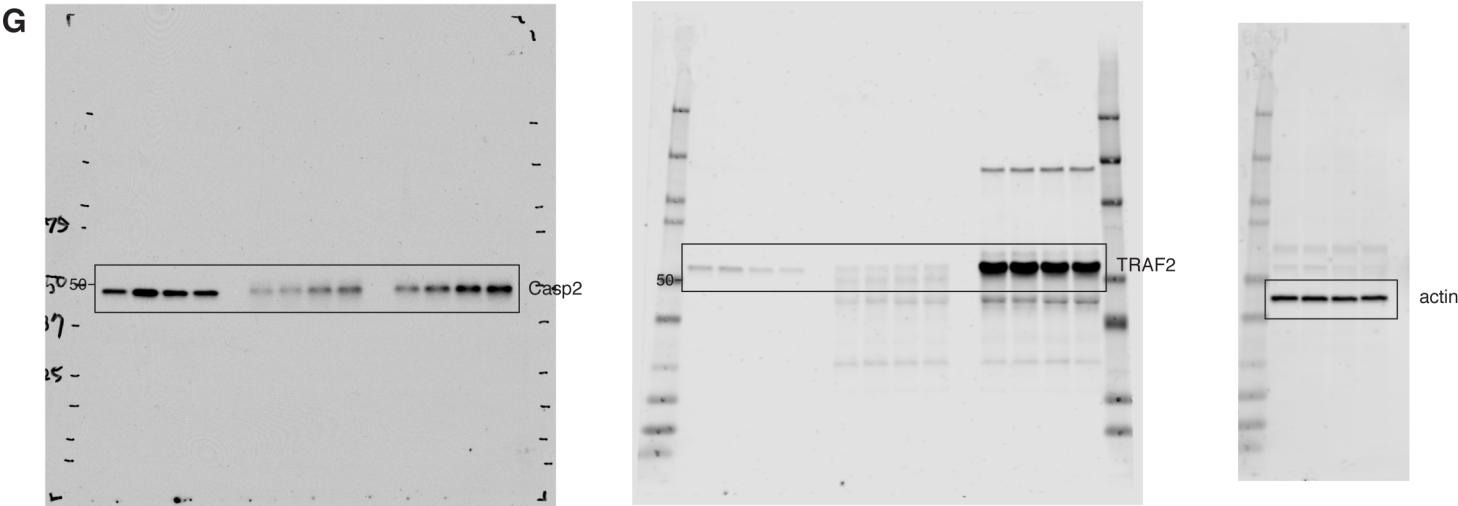

Supplement: Supplementary file 5 — Source Data for Figure 2 [file EMBJ-37-e97072-s003.pdf]

Figure 3

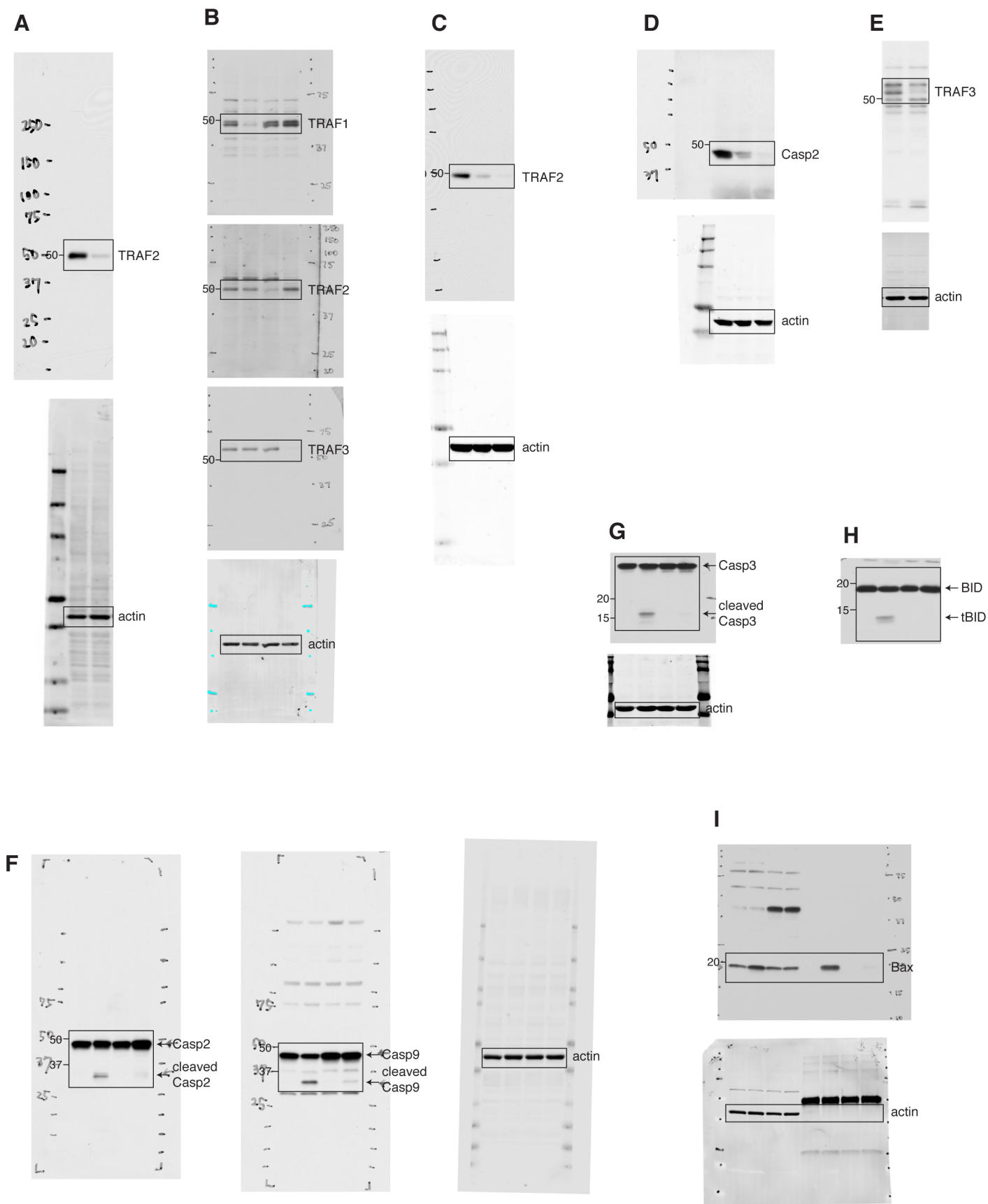

Supplement: Supplementary file 6 — Source Data for Figure 3 [file EMBJ-37-e97072-s004.pdf]

Figure 4

B

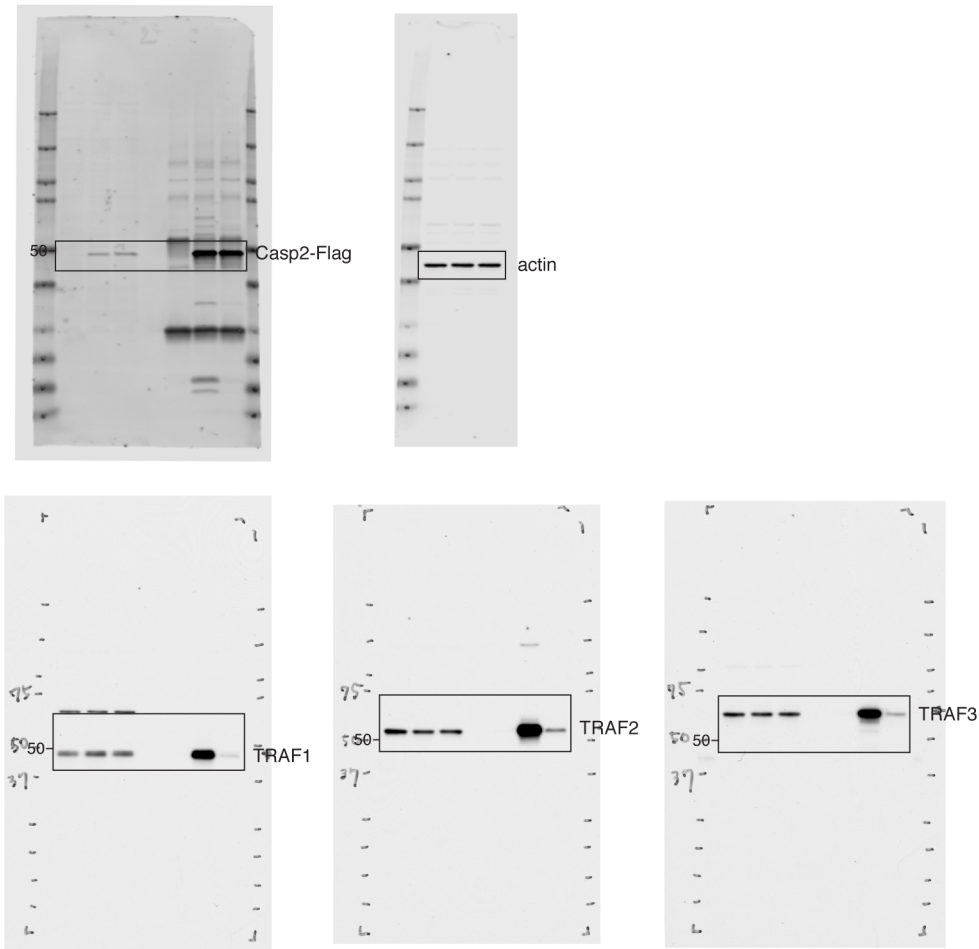

C

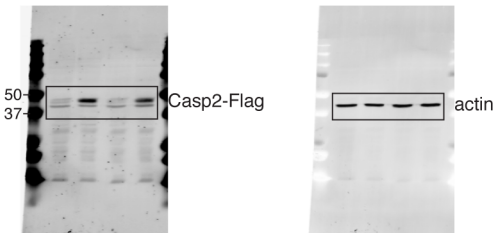

Supplement: Supplementary file 7 — Source Data for Figure 4 [file EMBJ-37-e97072-s005.pdf]

Figure 5

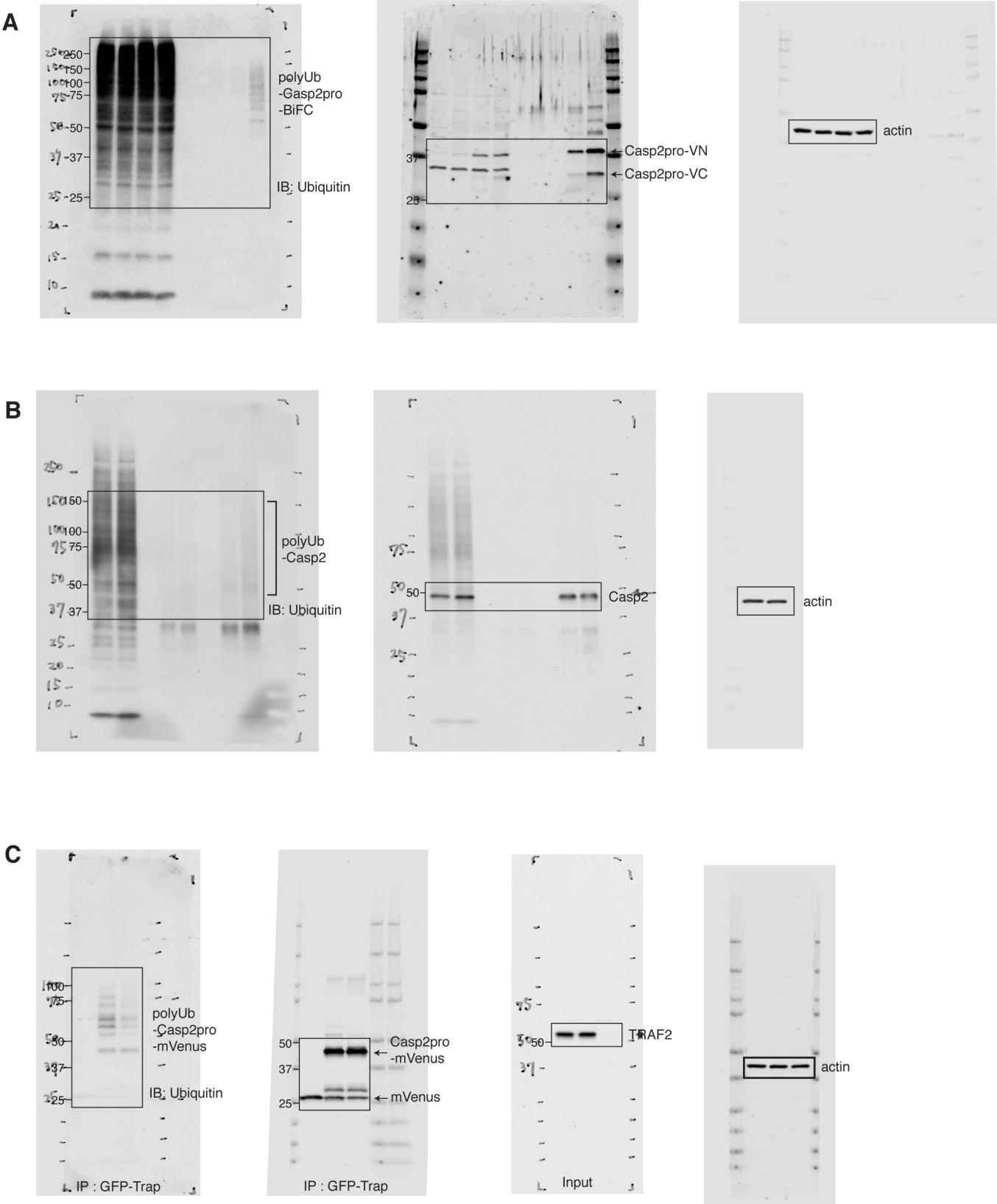

Figure 5

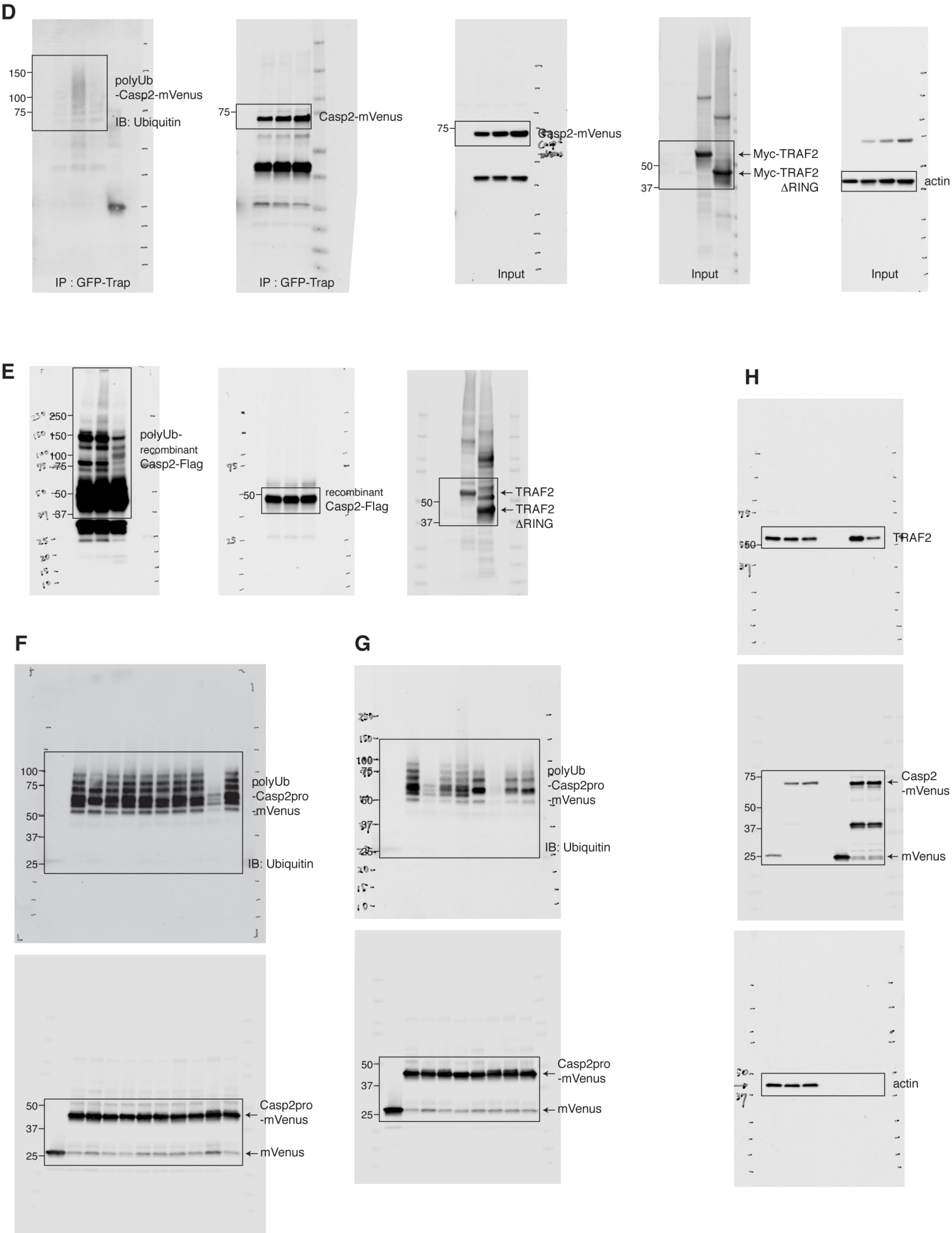

Figure 5

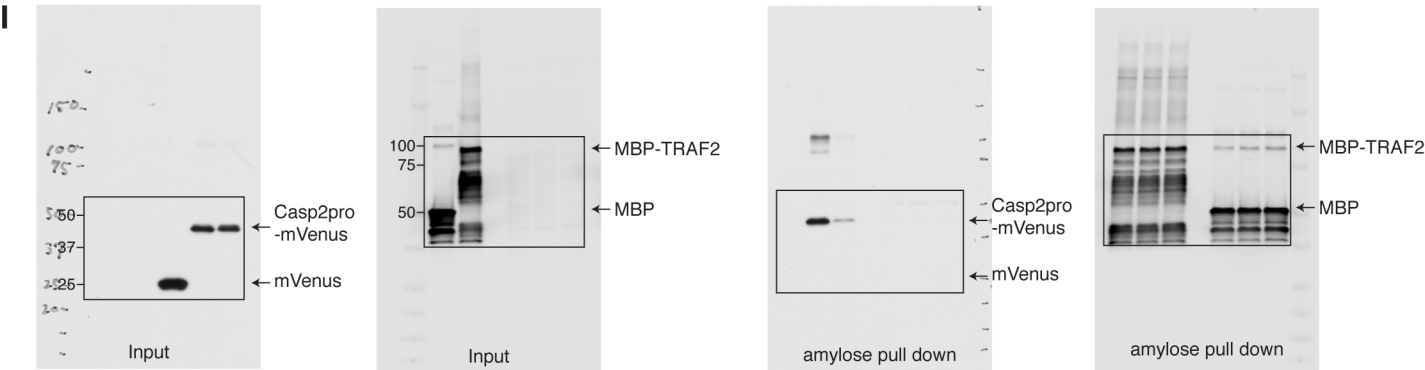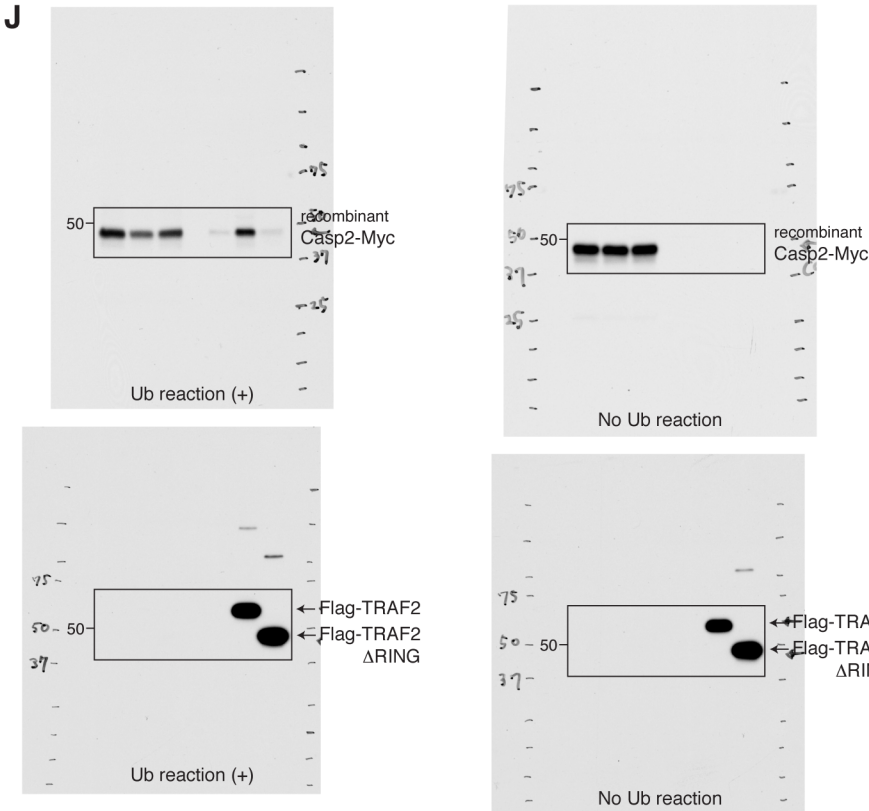

Supplement: Supplementary file 8 — Source Data for Figure 5 [file EMBJ-37-e97072-s006.pdf]

Figure 6

B

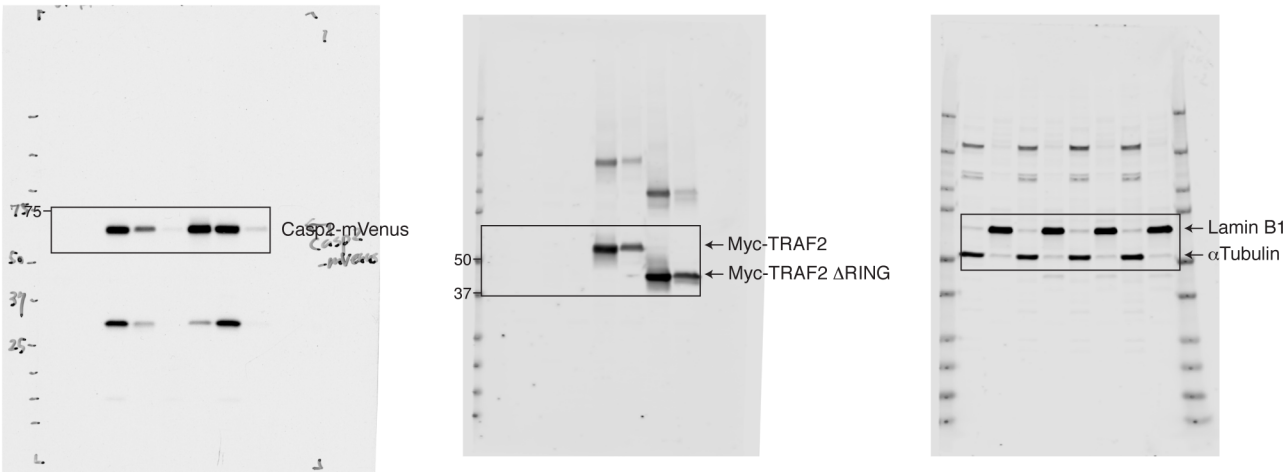

D

C

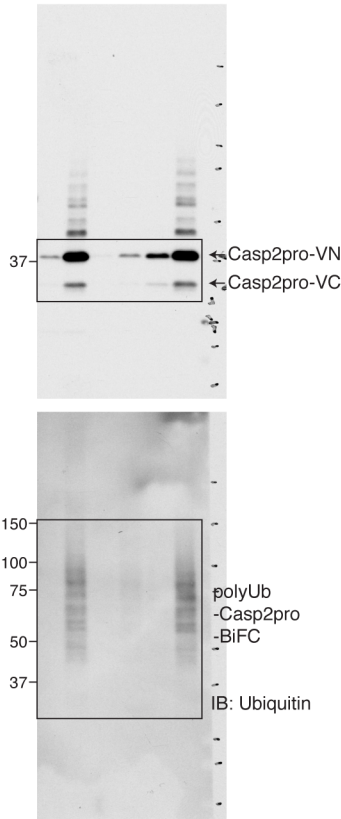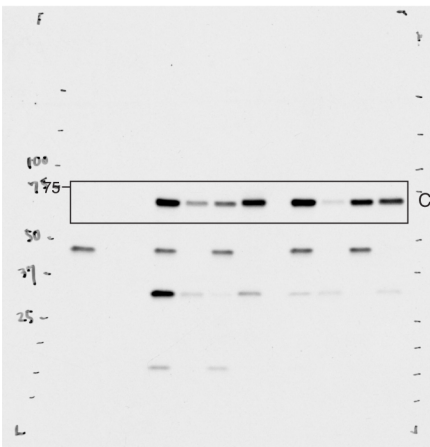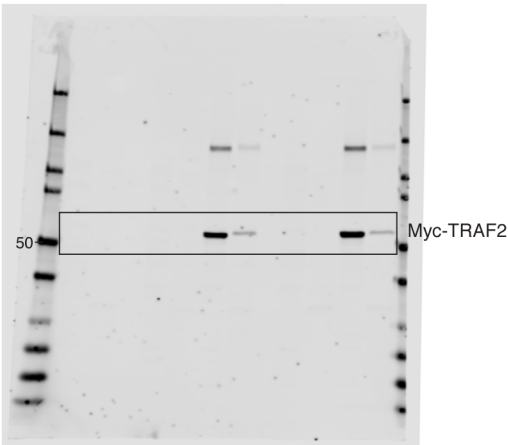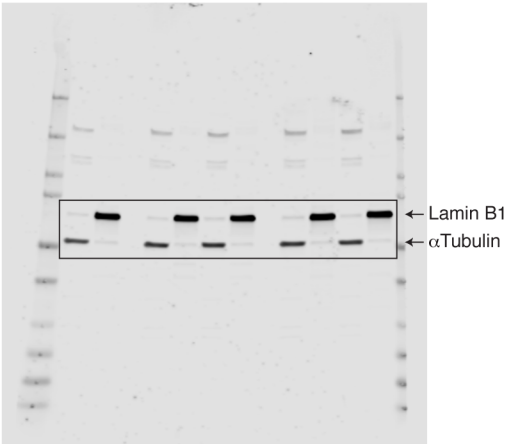

E

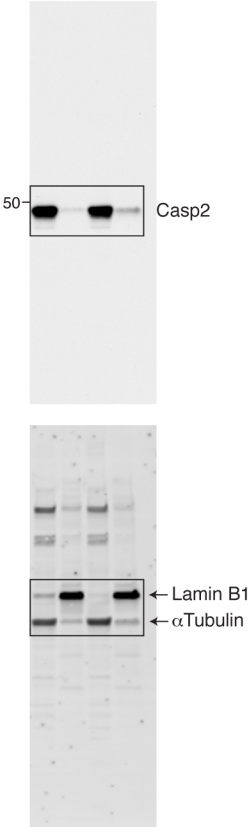

Supplement: Supplementary file 9 — Source Data for Figure 6 [file EMBJ-37-e97072-s007.pdf]

Figure 7

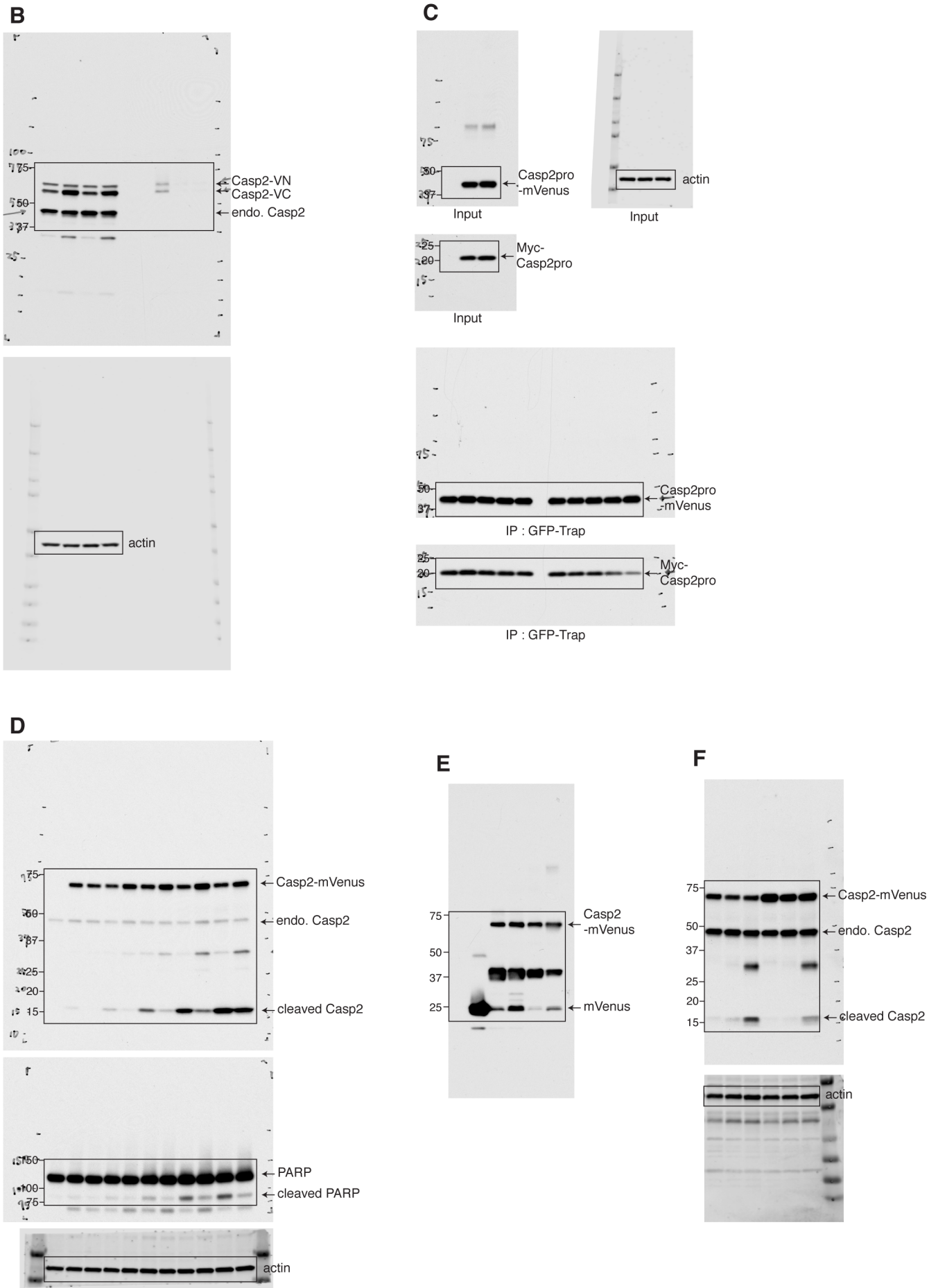

Supplement: Supplementary file 10 — Source Data for Figure 7 [file EMBJ-37-e97072-s008.pdf]
